# Disaster-related healthcare system disruption and maternal–neonatal outcomes

**DOI:** 10.3389/fpubh.2026.1784853

**Published:** 2026-05-29

**Authors:** Emrullah Akay, Ahmet Çinar, Ayşe Tuzcu, Alime Dilayda Uzun Gul¨, Lokman Semih Demirkaya

**Affiliations:** 1 Department of Obstetrics and Gynecology, Basaksehir Cam and Sakura City Hospital, Istanbul, Türkiye; 2 Department of Obstetrics and Gynecology, Hassa State Hospital, Hatay, Türkiye

**Keywords:** disaster-related health disparities, healthcare access, maternal health, maternal stress, perinatal outcomes, regional inequalities

## Abstract

**Introduction:**

Natural disasters disrupt healthcare systems and create crises that severely impact maternal and perinatal health outcomes. The 2023 Kahramanmaraş earthquakes caused widespread destruction in southeastern Turkey, leading to marked inequalities in healthcare access and obstetric service delivery. This study compared maternal and neonatal outcomes under disaster-related healthcare system disruption between an earthquake-affected region and an unaffected reference region.

**Methods:**

This retrospective comparative study included 403 women who delivered between February 6 and 21, 2023, at two hospitals located in an earthquake-affected region and an unaffected reference region. Maternal and neonatal outcomes were compared between the two settings. Continuous variables were analyzed using the independent samples *t*-test or Mann–Whitney *U*-test, and categorical variables using the chi-square or Fisher's exact test. Multivariable logistic regression analysis was performed to evaluate independent associations, and adjusted odds ratios (aOR) with 95% confidence intervals were reported.

**Results:**

The vaginal delivery rate was significantly higher and the cesarean section rate significantly lower in the earthquake-affected region (*p* < 0.05). Preterm birth (45.6%) and preterm premature rupture of membranes (7.4%) were more frequent in the affected group. Neonates born in the disaster-affected setting had significantly lower birth length and head circumference. Advanced cervical dilation and labor pain at admission were more common in the earthquake-affected group. Incomplete uterine rupture (uterine dehiscence) occurred more frequently compared with the control group (24.5% vs. 6.0%), while no complete uterine rupture was observed. Bilateral tubal ligation rates were markedly higher in the disaster-affected region (43.1%).

**Discussion:**

Significant disparities in maternal and neonatal health outcomes were identified in the context of disaster-related healthcare system disruption. These findings highlight the importance of healthcare system resilience, continuity of essential obstetric services, and preparedness strategies in post-disaster settings. This study provides real-world evidence of access inequalities following a major disaster, with implications for healthcare service planning, disaster preparedness, and public health policy.

## Background

Natural disasters profoundly disrupt social, economic, and healthcare systems worldwide, leading to both immediate harm and long-term public health consequences. Disruptions in healthcare services following disasters disproportionately affect socioeconomically vulnerable populations. Pregnant women, due to the physiological demands of pregnancy and barriers to healthcare access, represent one of the most vulnerable groups in disaster settings (1).

Pregnancy is a sensitive period, significantly influenced by physiological, psychosocial, and environmental factors. Limited access to healthcare during disasters increases the risks of adverse outcomes, such as preterm birth, low birth weight, and neonatal mortality (2). Additionally, disaster-induced stress and trauma negatively affect maternal mental health, further complicating pregnancy and postpartum recovery (3, 4). These challenges are exacerbated by regional disparities in healthcare access, contributing to inequalities in maternal and perinatal outcomes (5).

The 2023 Kahramanmaraş earthquakes caused extensive destruction in southeastern Turkey, severely disrupting healthcare infrastructure and exacerbating inequalities in maternal and perinatal health outcomes (6). The psychological effects of disasters during pregnancy not only influence birth outcomes but also pose significant threats to maternal mental health (7, 8). Elevated levels of maternal anxiety, depression, and post-traumatic stress disorder (PTSD) after such disasters highlight critical health disparities affecting both mothers and newborns (9, 10).

While existing studies have examined the impact of healthcare disruptions on maternal and neonatal health, limited attention has been paid to the role of socioeconomic and geographical disparities in disaster-affected regions. Research from the Haiti and Nepal earthquakes revealed that regional differences in healthcare access contributed to higher rates of preterm birth, low birth weight, and neonatal complications (11, 12). These findings underscore the importance of understanding post-disaster inequalities to inform policy development.

This study evaluates maternal and perinatal health outcomes at Dörtyol State Hospital, a region severely affected by the 2023 Kahramanmaraş earthquakes, and compares them with outcomes from a hospital in an unaffected region. Through this comparative approach, the study aims to highlight healthcare access disparities and system-level challenges in post-disaster settings. The findings provide evidence to inform future research and support the development of evidence-based policies to reduce maternal and perinatal health inequalities following disasters.

## Methods

### Study design

This retrospective comparative study was conducted using data obtained from the Obstetrics and Gynecology Clinic of Hatay Dörtyol State Hospital, one of the regions most severely affected by the two major earthquakes centered in Kahramanmaraş on February 6, 2023. As a comparison group, births from Istanbul Başakşehir Çam and Sakura City Hospital, located in a region unaffected by the earthquakes, were included.

### Study setting

#### Hatay Dörtyol state hospital

Hatay Dörtyol State Hospital is a secondary-level public hospital serving an earthquake-affected region. Prior to the earthquakes, the annual number of deliveries was approximately 500. Following the disaster, the hospital became a primary provider of obstetric care due to severe damage to other regional healthcare facilities, resulting in a substantial increase in delivery volume during the study period.

#### Çam and Sakura city hospital

Çam and Sakura City Hospital is a high-capacity tertiary referral center with an annual delivery volume of approximately 15,000. It was selected as the comparison center because it is located in a region unaffected by the earthquakes and provides comprehensive obstetric services comparable in clinical standards.

### Sampling method

All consecutive births occurring between February 6 and February 21, 2023, at both Hatay Dörtyol State Hospital and Çam and Sakura City Hospital were included in the study. Rather than using a specific sampling strategy, the study encompassed all births during this period to ensure a comprehensive and representative analysis.

### Study population

The study included a total of 403 pregnant women who gave birth at the two hospitals during the specified timeframe. At Hatay Dörtyol State Hospital, there were 97 vaginal deliveries, 101 cesarean deliveries, and 5 out-of-hospital deliveries (in an ambulance, field hospital, or at home). In comparison, Çam and Sakura City Hospital recorded 95 vaginal deliveries and 104 cesarean deliveries.

### Inclusion and exclusion criteria

- **Inclusion criteria**: The study included all pregnancies beyond 24 weeks of gestation that resulted in delivery. Both live births and stillbirths were analyzed. Having complete medical records was a prerequisite for inclusion.
- **Exclusion criteria:** Pregnancies under 24 weeks of gestation (early pregnancy loss or termination) and pregnancies that did not result in delivery were excluded. Furthermore, 2 patients who were referred to other facilities due to maternal or fetal complications in the earthquake-affected area and 3 patients with incomplete medical records were not included. Hence, a total of 5 patients were excluded.

The two referred patients required transfer for advanced care under emergency conditions during the disaster period. Due to limitations in follow-up and inter-institutional data access, definitive outcome data could not be obtained. Therefore, the potential impact of these cases on rare but severe outcomes cannot be fully excluded. However, the exclusion of these patients may introduce potential selection bias, particularly for rare and severe outcomes.

Additional clinical details regarding these patients are provided in Supplementary Table 1.

### Data collection

Clinical data from the earthquake-affected region and the unaffected reference center were obtained from hospital medical records and electronic hospital information systems. Demographic characteristics, delivery outcomes, maternal complications, neonatal parameters, and length of hospital stay were extracted and analyzed.

Data quality was ensured through standardized data extraction procedures and cross-checking between centers. All variables were defined prior to analysis, and consistency of measurements across centers was maintained.

### Variables

Collected variables included maternal demographic characteristics (age, gravidity, parity), mode of delivery, maternal complications, neonatal outcomes (birth weight, length, head circumference, and APGAR scores), and duration of hospital stay.

The primary independent variable was region (earthquake-affected vs. non-affected). Dependent variables included maternal outcomes such as preterm birth, PPROM, uterine dehiscence, and mode of delivery, as well as neonatal outcomes including birth weight, length, head circumference, and APGAR scores.

### Evaluation criteria

- **Preterm Birth**: Defined as delivery before 37 weeks of gestation. This parameter was considered a critical indicator of neonatal morbidity and mortality.
  Early preterm: < 34 weeks
  Late preterm: 34–36+6 weeks
  Term: ≥37 weeks
- **Low Birth Weight**: Defined as a neonatal birth weight below 2,500 grams.
- **Postpartum Hemorrhage (PPH)**: Defined as blood loss exceeding 500 mL after vaginal delivery or 1,000 mL after cesarean section.
- **Preterm Premature Rupture of Membranes (PPROM)**: Defined as spontaneous rupture of amniotic membranes before 37 weeks of gestation.
- **Perineal Injury**: Defined as anal sphincter rupture or severe perineal tissue damage following vaginal delivery.
- **Incomplete Uterine Rupture (Uterine Dehiscence)**: Defined as an incomplete separation of the uterine wall, usually at the site of a previous cesarean scar, with intact serosa and without full-thickness disruption or fetal extrusion into the peritoneal cavity.
  No cases of complete uterine rupture were identified in the study population.
- **Apgar Score**: Defined as a numerical assessment of neonatal wellbeing at 1 and 5 min after birth, with scores below 7 indicating potential complications.
- **Bilateral Tubal Ligation (BTL)**: Defined as a permanent surgical sterilization procedure involving the occlusion or removal of the fallopian tubes, typically performed to prevent future pregnancies.
- **Fetal Distress**
  Fetal distress was defined based on the presence of one or more of the following clinical and electronic fetal monitoring criteria:
  1. Persistent fetal bradycardia (< 110 bpm) or tachycardia (>160 bpm),
  2. Repetitive late or severe variable decelerations on cardiotocography,
  3. Absence of variability in the fetal heart rate.

### Statistical analysis

Statistical analyses were conducted using IBM SPSS Statistics version 27.0 (IBM Corp., Armonk, NY, USA). Continuous variables were assessed for normality using the Kolmogorov–Smirnov and Shapiro–Wilk tests. Normally distributed variables were presented as mean ± standard deviation, while non-normally distributed variables were expressed as median (interquartile range). Categorical variables were summarized as counts and percentages.

For univariate comparisons, the independent samples *t*-test was used for normally distributed variables and the Mann–Whitney *U*-test for non-normally distributed variables. Categorical variables were compared using the chi-square test or Fisher's exact test, as appropriate.

To account for potential confounding and to evaluate independent associations, multivariable logistic regression analyses were performed. Adjusted odds ratios (aOR) with 95% confidence intervals (CI) were calculated. The models were adjusted for clinically relevant confounders, including maternal age and parity.

Subgroup analyses were conducted according to gestational age categories (early preterm, late preterm, and term) to further assess neonatal outcomes.

To account for multiple comparisons, the Benjamini–Hochberg false discovery rate (FDR) correction was applied to the primary outcomes presented in Tables 2, 3, and adjusted *p*-values are reported.

All statistical tests were two-tailed, and a *p*-value of < 0.05 was considered statistically significant.

## Results

### Participant distribution

A total of 403 pregnant women were included in the study. Of these, 204 (50.6%) delivered at Hatay Dörtyol State Hospital and 199 (49.4%) at Istanbul Başakşehir Çam and Sakura City Hospital.

### Demographic data

The mean age of patients in the earthquake-affected region (26.8 ± 6.4 years) was significantly lower than in the Istanbul group (29.0 ± 5.5 years, *p* < 0.05). No significant difference was observed in the number of gravidities between groups (*p* > 0.05). However, the multiparity rate in the earthquake-affected group (91.7%) was significantly higher than in the Istanbul group (68.8%; *p* < 0.05).

Additionally, the number of living children was higher in the earthquake-affected group, whereas the Istanbul group had a significantly higher abortion rate (*p* < 0.05). No significant difference was noted in the number of miscarriages (*p* > 0.05). The proportions of patients with a history of cesarean delivery or uterine surgery were similar between groups (*p* > 0.05) (Table 1).

**Table 1:** Baseline demographic and obstetric characteristics of women in earthquake-affected and unaffected regions.

Variable	Category	Istanbul city hospital group (*n* = 199)				Earthquake zone group (*n* = 204)				*p*-value
		Mean ±SD/n (%)			Median	Mean ±SD/n (%)			Median	
Age		29.0	±	5.5	29.0	26.8	±	6.4	27.0	**< 0.001**
Gravida Number		2.6	±	1.5	2.0	2.8	±	1.8	2.0	0.335
Parity	0	62		31.2%		17		8.3%		**< 0.001**
	≥1	137		68.8%		187		91.7%		
Parity (mean)		1.8	±	1.0	2.0	2.0	±	1.2	2.0	0.550
Living Child	0	65		32.7%		57		27.9%		0.302
	≥1	134		67.3%		147		72.1%		
Living children (mean)		1.8	±	1.0	2.0	2.1	±	1.2	2.0	**0.048**
Abortus	0	148		74.4%		170		83.3%		**0.027**
	≥1	51		25.6%		34		16.7%		
Aborts (mean)		1.5	±	0.8	1.0	1.7	±	1.2	1.0	0.950
History of cesarean section or uterine surgery	0	147		73.9%		153		75.0%		0.795
	≥1	52		26.1%		51		25.0%		

Data are presented as number (%) or mean ± standard deviation (median). Statistical analyses were performed using the Chi-square test or Mann–Whitney U-test, as appropriate. Bold values indicate statistically significant results (*p* < 0.05).

### Maternal findings

**Figure 1:**
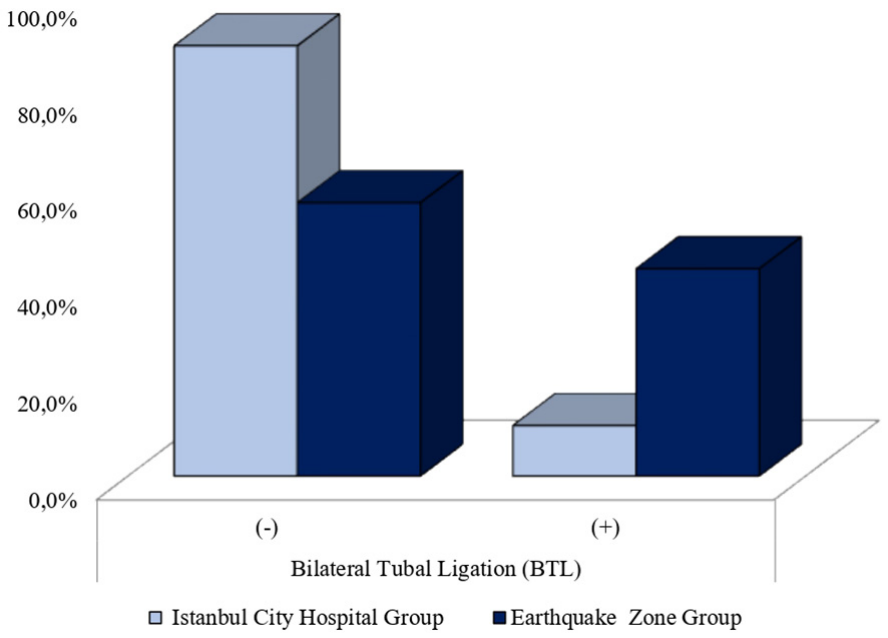
Bilateral tubal ligation (BTL) rates: disaster region vs. control group.

The rate of vaginal delivery was significantly higher in the earthquake-affected region, whereas the cesarean section rate was significantly lower. Cervical dilation at admission was greater in the earthquake-affected group, and labor pain at admission was more frequent. The mean gestational age was lower in the earthquake-affected group compared with the Istanbul group (Figure 1; Table 2).

**Table 2:** Comparison of maternal outcomes between earthquake-affected and unaffected regions.

Variable	Category	Istanbul city hospital group (*n* = 199)				Earthquake zone group (*n* = 204)				*p*-value	Adjusted *p*-value (FDR)
		Mean ±SD/n (%)			Median	Mean ±SD/n (%)			Median		
Mode of delivery	Cesarean section	133		66.8%		102		50.0%		**0.001**	**0.012**
	Vaginal delivery	66		33.2%		102		50.0%			
Presence of labor pain at admission		101		50.8%		159		77.9%		**< 0.001**	**0.006**
Cervical dilation at admission (cm)		3.8	±	2.5	4.0	6.8	±	2.0	7.0	**< 0.001**	**0.005**
Gestational age (weeks)		38.2	±	2.6	38.6	37.3	±	2.4	37.1	**< 0.001**	**0.004**
Preterm delivery		41		20.6%		93		45.6%		**< 0.001**	**0.0036**
Preterm premature rupture of membranes (PPROM)		4		2.0%		15		7.4%		**0.011**	**0.017**
Fetal distress		33		16.6%		9		4.4%		**< 0.001**	**0.0031**
Postpartum hemorrhage (PPH)		19		9.5%		24		11.8%		0.471	0.490
Perineal injury		4		6.1%		10		9.8%		0.391	0.425
Bilateral tubal ligation (BTL)		14		10.5%		44		43.1%		**< 0.001**	**0.0028**
Incomplete uterine rupture (Uterine Dehiscence)		8		6.0%		25		24.5%		**< 0.001**	**0.0025**
Length of hospital stay (days)		2.2	±	1.7	2.0	3.3	±	1.2	3.0	**< 0.001**	**0.0023**
Length of hospital stay (days)	Cesarean section	2.50	±	1.6	2.0	3.40	±	1.15	3.0	**< 0.001**	**0.003**
	Vaginal delivery	1.67	±	1.7	1.0	3.22	±	1.32	3.0		**0.0035**

Data are presented as number (%) or mean ± standard deviation (median). Statistical analyses were performed using the Chi-square test or Mann–Whitney *U*-test, as appropriate. The Benjamini–Hochberg false discovery rate (FDR) correction was applied, and adjusted *p*-values are reported. A false discovery rate of 5% was considered statistically significant. *P*-values < 0.001 were treated as 0.001 for adjustment.

Preterm birth rates were higher in the earthquake-affected region (*p* < 0.05). The incidence of PPROM was also higher in the earthquake-affected group than in the Istanbul group (*p* < 0.05). Fetal distress was more frequent in the Istanbul group, with 9 (4.4%) cases in the earthquake-affected region and 33 (16.6%) cases in the Istanbul group (*p* < 0.001) (Table 2).

**Figure 2:**
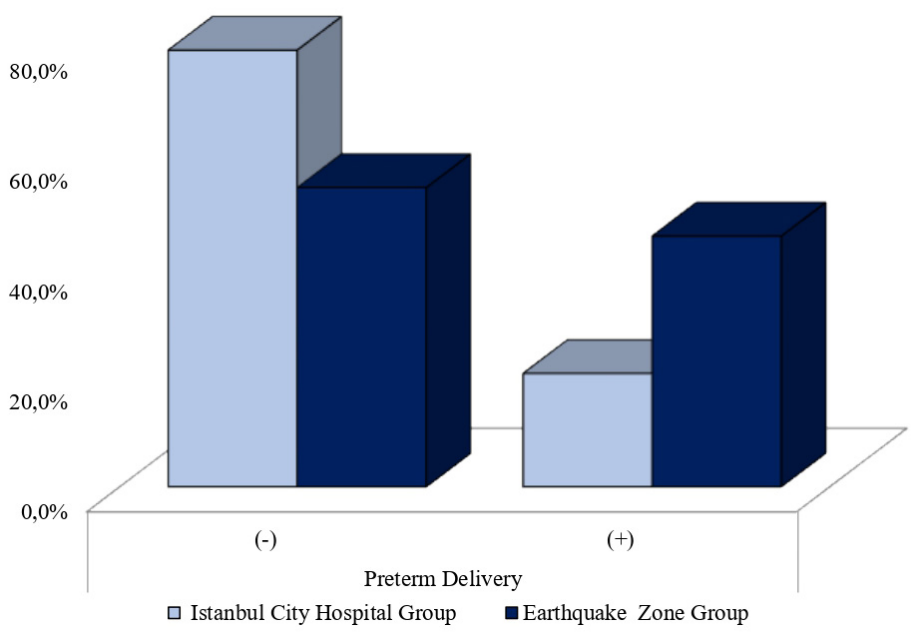
Preterm birth rates: disaster-affected vs. control regions.

**Figure 3:**
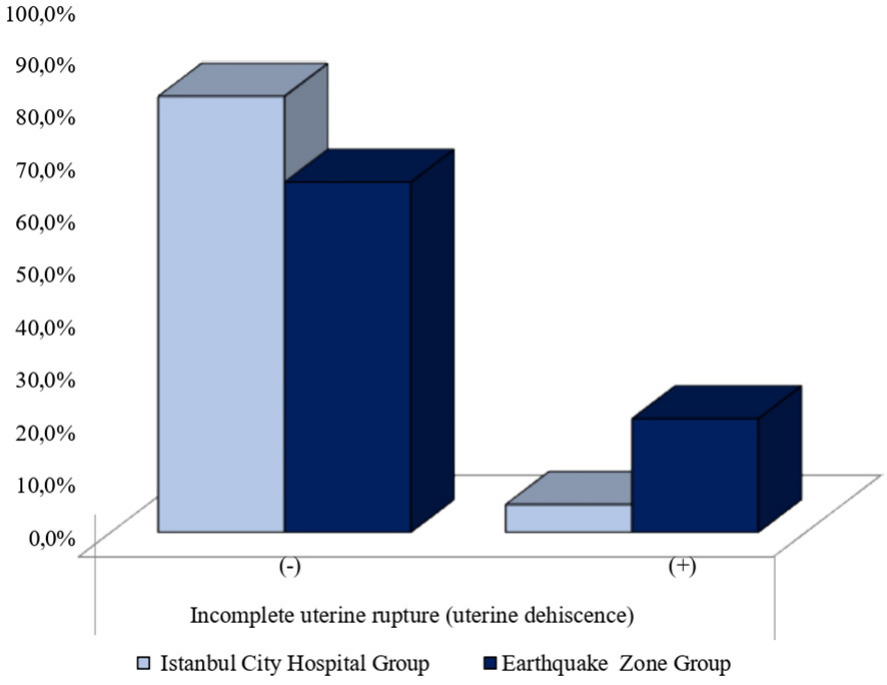
Incomplete uterine rupture (Uterine Dehiscence) rates: disaster region vs. control group.

The rate of incomplete uterine rupture (uterine dehiscence) was significantly higher in the earthquake-affected region compared with the control group (24.5% vs. 6.0%, *p* < 0.001). No cases of complete uterine rupture were observed in either group (Figure 2). The rate of bilateral tubal ligation was significantly higher in the earthquake-affected region (Figure 3).

Hospital stay was significantly longer in the earthquake-affected group compared with the Istanbul group (mean ± SD: 3.3 ± 1.2 days, median 3.0 days vs. 2.2 ± 1.7 days, median 2.0 days; *p* < 0.001). When stratified by mode of delivery, median hospital stay following vaginal delivery was 3.0 days in the earthquake-affected group and 1.0 day in the Istanbul group. For cesarean delivery, median hospital stay was 3.0 days in the earthquake-affected group and 2.0 days in the Istanbul group (*p* < 0.001) (Table 2).

### Fetal outcomes

No significant differences were observed in birth weight between the two groups (*p* > 0.05). Birth length and head circumference were significantly lower in the earthquake-affected region compared with the Istanbul group (49.6 ± 2.3 cm vs. 50.9 ± 3.8 cm and 34.7 ± 1.3 cm vs. 34.9 ± 2.4 cm, respectively; *p* < 0.05). The 1-min APGAR scores were significantly lower in the earthquake-affected group (*p* < 0.05), while the 5-min APGAR scores were significantly higher (*p* < 0.05). There was no significant difference between groups in terms of neonatal sex distribution (*p* > 0.05) (Table 3).

**Table 3:** Comparison of fetal outcomes between Istanbul city hospital and earthquake zone groups.

Variable	Category	Istanbul city hospital group (*n* = 199)				Earthquake zone group (*n* = 204)				*p*-value	Adjusted *p*-value (FDR)
		Mean ±SD/n (%)			Median	Mean ±SD/n (%)			Median		
Birth weight (g) overall		3,113.1	±	671.7	3,210.0	3,178.7	±	469.2	3,135.0	0.902	0.902
Birth weight (g)	Early preterm	2,038	±	415	2,010	1,776	±	404	1,716	0.042	0.070
	Late preterm	2,805	±	331	2,838	2,649	±	285	2,660	0.034	0.068
	Term	3,302	±	620	3,303	3,171	±	506	3,167	0.126	0.158
Birth length (cm)		50.9	±	3.8	52.0	49.6	±	2.3	50.0	**< 0.001**	**0.004**
Head circumference (cm)		34.9	±	2.4	35.0	34.7	±	1.3	35.0	**0.006**	**0.012**
Head circumference (cm)	Early preterm	29.1	±	2.7	28.8	27.8	±	1.7	27.7	0.050	0.071
	Late preterm	33.0	±	1.4	32.8	32.2	±	1.7	32.1	0.048	0.072
	Term	34.9	±	1.5	35.0	34.1	±	1.6	34.2	**< 0.001**	**0.004**
Baby gender	Female	87		43.9%		104		51.0%		0.158	0.176
	Male	111		56.1%		100		49.0%			
**APGAR score**											
**At 1 min**		6.0	±	1.8	6.0	5.7	±	2.1	6.0	**< 0.001**	**0.004**
**At 5 min**		8.6	±	1.7	9.0	9.0	±	1.1	9.0	**< 0.001**	**0.004**

Data are presented as number (%) or mean ± standard deviation (median). Statistical analyses were performed using the Chi-square test or Mann–Whitney *U*-test, as appropriate. The Benjamini–Hochberg false discovery rate (FDR) correction was applied, and adjusted *p*-values are reported. A false discovery rate of 5% was considered statistically significant. *P*-values < 0.001 were treated as 0.001 for adjustment.

### Subgroup analysis by gestational age

Birth weight and head circumference were analyzed according to gestational age categories. In the earthquake-affected group, both early preterm (< 34 weeks) and late preterm (34–36+6 weeks) neonates had significantly lower mean birth weight and head circumference values compared with the corresponding gestational age categories in the Istanbul group (*p* < 0.05). At term (≥37 weeks), head circumference was also significantly lower in the earthquake-affected group (34.1 ± 1.6 cm vs. 34.9 ± 1.5 cm, *p* < 0.05) (Table 3).

After adjustment for multiple comparisons, only selected outcomes remained statistically significant (Tables 2, 3).

Multivariable logistic regression analysis showed that delivery in the earthquake-affected region was independently associated with preterm birth (adjusted OR 3.69, 95% CI 2.27–6.00; *p* < 0.001), PPROM (adjusted OR 4.57, 95% CI 1.37–15.17; *p* = 0.013), and incomplete uterine rupture (adjusted OR 6.06, 95% CI 2.47–14.88; *p* < 0.001) (Table 4).

**Table 4:** Multivariable logistic regression analysis of maternal outcomes.

Outcome	Variable	Adjusted OR (95% CI)	*p*-value
Preterm birth	Earthquake-affected region	**3.69 (2.27–6.00)**	< 0.001
	Age (years)	0.98 (0.94–1.02)	0.310
	Parity	1.12 (0.90–1.40)	0.280
PPROM	Earthquake-affected region	**4.57 (1.37–15.17)**	0.013
	Age (years)	1.01 (0.95–1.08)	0.640
	Parity	1.05 (0.78–1.42)	0.740
Incomplete uterine rupture (uterine dehiscence)	Earthquake-affected region	**6.06 (2.47–14.88)**	< 0.001
	Age (years)	1.02 (0.96–1.08)	0.410
	Parity	1.18 (0.85–1.64)	0.310

Multivariable logistic regression analysis was performed. Models were adjusted for age and parity. Odds ratios (ORs) with 95% confidence intervals (CIs) are presented. The reference category was the non-affected region.

The association between prolonged hospital stay and clinical complications was statistically significant (*p* < 0.001) (Table 5).

**Table 5:** Association between prolonged hospital stay and clinical complications in the earthquake-affected group.

Hospital stay	Complication (-)	Complication (+)	Total	*p*-value
≤ 3 days	148	35	183	**< 0.001**
>3 days	6	15	21	

Statistical analysis was performed using the chi-square or Fisher's exact test, as appropriate. A *p*-value < 0.05 was considered statistically significant.

## Discussion

The present study provides comparative real-world evidence on maternal and perinatal outcomes in the context of disaster-related healthcare system disruption following the February 2023 Kahramanmaraş earthquakes. By comparing an earthquake-affected region with an unaffected reference setting, our findings indicate that large-scale emergencies are associated with measurable differences in obstetric and neonatal outcomes. Importantly, these associations remained significant after adjustment for maternal age and parity, suggesting that the observed differences are not fully explained by baseline demographic characteristics.

Natural disasters have been associated with adverse maternal and neonatal outcomes, particularly an increased risk of preterm birth. In the present study, preterm birth rates were higher in the earthquake-affected group compared with the control group (*p* < 0.05). Maternal stress related to disaster exposure has been suggested as a potential contributing factor to early delivery, consistent with previous disaster-related studies reporting higher rates of preterm birth in such settings (13–15).

Previous studies conducted after large-scale disasters have reported that effective interventions and well-organized healthcare systems are associated with more favorable perinatal outcomes, with preterm birth rates remaining comparable to national averages (16). These findings suggest that continuity of prenatal care and supportive healthcare conditions may play a role in maternal and neonatal outcomes in disaster settings.

Consistent with these observations, subgroup analysis based on gestational age showed that preterm infants in the earthquake-affected region had lower birth weights and head circumferences than those in the Istanbul group. These differences were also observed among term deliveries, indicating that factors beyond prematurity may contribute to differences in neonatal growth in disaster-affected settings (17).

In this study, the incidence of PPROM was higher in the disaster-affected region than in the control group (*p* < 0.05). Limited access to healthcare and resource constraints may contribute to this finding. In disaster settings, delays in care may complicate PPROM management, while healthcare system disruptions may limit the effectiveness of appropriate obstetric interventions (13–15, 17).

The higher rate of vaginal delivery and lower rate of cesarean section observed in the disaster-affected region (*p* < 0.05) may reflect differences in access to surgical care during the disaster period. Damage to healthcare infrastructure and shortages of trained personnel may affect cesarean delivery capacity in emergency settings. Previous disaster-related studies have reported similar patterns, while settings with well-organized and resilient healthcare systems have reported stable or even increased cesarean section rates during disasters (17–20).

Turkey's high baseline cesarean section rate, which exceeds global averages (21, 22), may be associated with the elevated cesarean rates observed in unaffected regions. The lower cesarean rate in the disaster-affected area may reflect constraints in surgical capacity rather than differences in obstetric risk profiles. These findings suggest that disaster-related system limitations may influence delivery mode patterns and indicate the need to maintain surgical capacity for high-risk pregnancies in post-disaster settings.

Incomplete uterine rupture was more frequent in the earthquake-affected region compared with the control group (*p* < 0.05). Although the literature directly examining the association between earthquakes and uterine dehiscence is limited, disaster-related factors such as limited healthcare resources, delayed hospital admission, and staff shortages may contribute to this finding (23, 24). This observation supports the relevance of maintaining intrapartum monitoring capacity and referral pathways in disaster preparedness strategies.

Lower 1-min APGAR scores were observed in the earthquake-affected group (*p* < 0.05), while 5-min APGAR scores were higher. Previous studies have reported increased rates of low birth weight, preterm birth, and lower APGAR scores following severe disasters (25, 26). These findings suggest that intrapartum and early neonatal care conditions may influence neonatal outcomes in disaster settings.

Fewer cases of fetal distress were reported in the earthquake-affected region. This finding may be influenced by differences in diagnostic capacity and clinical conditions between centers, including availability of monitoring resources and timing of hospital presentation (25, 26). In addition, advanced cervical dilation at admission may complicate the assessment of fetal wellbeing.

In the earthquake-affected group, advanced cervical dilation (*p* < 0.05) and severe labor pain at admission (*p* < 0.05) were more frequent, which may be associated with delayed access to obstetric care. Infrastructure damage, staff shortages, and increased patient volume during the disaster period may have affected the management of obstetric complications. In disaster settings, healthcare delivery conditions may differ from standard clinical environments (27).

Hospital stay was longer in the earthquake-affected region. Median length of stay following vaginal delivery was 3.0 days compared with 1.0 day in the Istanbul group, while cesarean deliveries were associated with median stays of 3.0 and 2.0 days, respectively (*p* < 0.001).

To further clarify the determinants of prolonged hospitalization, we performed an additional analysis restricted to the earthquake-affected group. Prolonged hospital stay (>3 days) was significantly associated with clinical complications, including preterm birth, PPROM, and uterine dehiscence (*p* < 0.001; Table 5).

These findings suggest that extended hospitalization in the disaster-affected region was primarily driven by clinical factors. However, prolonged stays were not exclusively limited to patients with complications. Non-clinical factors such as post-disaster living conditions, housing loss, and limited access to safe discharge environments may also have contributed, as previously reported in disaster-related settings (28). Therefore, the contribution of social factors should be interpreted cautiously.

A higher rate of BTL was observed in the earthquake-affected region (*p* < 0.05). This finding may be associated with changes in reproductive preferences in the post-disaster context. Previous studies have reported declines in birth rates and shifts in reproductive behavior after major disasters (29–32). These findings suggest that access to family planning and counseling services may be relevant in post-disaster settings to support women's reproductive health and psychosocial well-being.

Major disasters have been associated with adverse effects on women's mental health, with potential implications for pregnancy outcomes. In the present study, lower APGAR scores were observed in the earthquake-affected group. Previous disaster-related studies have reported increased rates of depression, anxiety, and post-traumatic stress symptoms among affected women (33–37). Women with low socioeconomic status in severely impacted regions may be particularly vulnerable. These findings suggest that psychosocial support and mental health services may be relevant components of maternal care in disaster settings.

Continuity of perinatal services and timely organizational responses may play an important role in maternal and neonatal health in disaster settings. Evidence from previous disasters suggests that reorganization of healthcare infrastructure is associated with more favorable obstetric outcomes, whereas disruptions in service delivery may influence practices such as mode of delivery and breastfeeding (34, 38, 39). In this context, the present study supports the relevance of coordinated response mechanisms, efficient logistics, and the maintenance of essential reproductive health services in the aftermath of large-scale disasters.

These findings should be interpreted with caution due to the observational design of the study and the potential for residual confounding. Although multivariable analysis was performed, adjustment was limited to available variables, and unmeasured factors may have influenced the observed associations. Additionally, multiple statistical comparisons were addressed using the Benjamini–Hochberg false discovery rate (FDR) correction; however, residual risk of type I error (false-positive findings) cannot be completely excluded. Findings that did not remain statistically significant after adjustment should be interpreted with caution. Despite these limitations, the overall consistency of findings across multiple maternal and neonatal outcomes supports the robustness of the observed associations.

### Strengths and limitations

This study provides real-world evidence on maternal and neonatal outcomes in a post-disaster setting by comparing an earthquake-affected center with an unaffected reference hospital, thereby offering insight into healthcare service disruption in such settings.

Several limitations should be acknowledged. The absence of pre-disaster baseline data limits causal interpretation, and severe outcomes may have been underestimated due to excluded referrals and unmeasured barriers to healthcare access. In addition, the retrospective design and single-center nature of the disaster-affected cohort may limit generalizability.

Residual confounding due to unmeasured variables cannot be excluded. Furthermore, denominators for certain outcomes, such as uterine rupture and bilateral tubal ligation rates, should be interpreted with caution. Multiple comparisons were addressed using the Benjamini–Hochberg false discovery rate (FDR) correction; however, residual risk of type I error (false-positive findings) cannot be completely excluded.

## Conclusions

Large-scale disasters are associated with differences in maternal and neonatal outcomes, potentially related to healthcare system disruption and reduced service capacity. These findings suggest that strengthening disaster-resilient health systems, ensuring continuity of obstetric care, and integrating preparedness strategies into public health planning may be relevant for improving maternal and perinatal outcomes in future large-scale emergencies.

## Data Availability

The data analyzed in this study is subject to the following licenses/restrictions: The datasets are restricted due to patient confidentiality and are not publicly available under institutional and national data protection regulations. Requests to access these datasets should be directed to emreakaydr@hotmail.com.

## References

[1] Mesrkanlou HA Hezaveh SJG Tahmasebi S Nikniaz Z Nikniaz L. The effect of an earthquake experienced during pregnancy on maternal health and birth outcomes. Disaster Med Public Health Prep. (2022) 17:e157. doi: 10.1017/dmp.2022.132 35757895

[2] Asl YP Ghanbari-Homaie S Partash N. Consequences of natural and man-made disasters on pregnancy outcomes and complications: a systematic review. Arch Acad Emerg Med. (2024) 12:e61. doi: 10.22037/aaem.v12i1.2268 39296519 PMC11408978

[3] Dilcen HY Koçak YÇ Ada G Bozkurt FD. Determinants of psychosocial health status in pregnant and postpartum women experiencing earthquake in Turkey. Disaster Med Public Health Prep. (2024) 18:e16. doi: 10.1017/dmp.2024.11 38304943

[4] Qu Z Tian D Zhang Q Wang X Zhang L. The impact of the catastrophic earthquake in China's Sichuan province on the mental health of pregnant women. J Affect Disord. (2012) 136:117–23. doi: 10.1016/j.jad.2011.08.021 21937121

[5] Ahmed SK Khdhir RM. Protecting the health of pregnant women in Turkey and Syria earthquake-affected areas: challenges and opportunities. Womens Health. (2023) 19:17455057231166281. doi: 10.1177/17455057231166281 PMC10084526 37025059

[6] Topcu EG. Disaster preparedness: the effects of natural disasters on women's health in Turkey. Int J Gynaecol Obstet. (2023) 163:345–7. doi: 10.1002/ijgo.15149 37723975

[7] Ibici Akça E Gökbulut N Senoglu A. Pregnant women's depression and posttraumatic stress levels after the large-scale Turkey earthquakes: a cross-sectional study. Womens Health. (2024) 64:736–44. doi: 10.1080/03630242.2024.2402789 39261979

[8] Taner Derman M Türen S. Concerns of earthquake survivor mothers for their children and the role of school leadership in addressing them. Front Public Health. (2025) 13:1555125. doi: 10.3389/fpubh.2025.1555125 40880937 PMC12380865

[9] Chen XY Chen J Shi X Jiang M Li Y Zhou Y . Trajectories of maternal symptoms of posttraumatic stress disorder predict long-term mental health of children following the Wenchuan earthquake in China: a 10-year follow-up study. J Affect Disord. (2020) 266:201–6. doi: 10.1016/j.jad.2020.01.084 32056877

[10] Çinaroglu M Yilmazer E Noyan Ahlatcioglu E Ülker SV Hizli Sayar G. Psychological impact of the 2023 Kahramanmaraş earthquakes: a systematic review and meta-analysis of PTSD, depression, and anxiety among Turkish adults. Front Public Health. (2025) 13:1664212. doi: 10.3389/fpubh.2025.1664212 40933413 PMC12417135

[11] Harville EW Do M. Reproductive and birth outcomes in Haiti before and after the 2010 earthquake. Disaster Med Public Health Prep. (2016) 10:59–66. doi: 10.1017/dmp.2015.69 26055727

[12] Khatri GK Tran TD Baral S Fisher J. Experiences of an earthquake during pregnancy, antenatal mental health and infants' birthweight in Bhaktapur district, Nepal, 2015: a population-based cohort study. BMC Pregnancy Childbirth. (2020) 20:414. doi: 10.1186/s12884-020-03086-5 32689955 PMC7370411

[13] Palmeiro-Silva YK Orellana P Venegas P Monteiro L Varas-Godoy M Norwitz E . Effects of earthquake on perinatal outcomes: a Chilean register-based study. PLoS ONE. (2018) 13:e0191340. doi: 10.1371/journal.pone.0191340 29474413 PMC5825031

[14] Harville EW Xiong X Mattison DR. Disasters and perinatal health: a systematic review. Obstet Gynecol Surv. (2010) 65:713–28. doi: 10.1097/OGX.0b013e31820eddbe 21375788 PMC3472448

[15] Lian Q Ni J Zhang J Zhang L. Maternal exposure to Wenchuan earthquake and prolonged risk of offspring birth outcomes: a natural experiment study. BMC Pregnancy Childbirth. (2020) 20:552. doi: 10.1186/s12884-020-03206-1 32962638 PMC7510090

[16] Kyozuka H Fujimori K Hosoya M Yasumura S Yokoyama T Sato A . The Japan environment and children's study (JECS) in Fukushima prefecture: pregnancy outcomes after the Great East Japan Earthquake. Tohoku J Exp Med. (2018) 246:27–33. doi: 10.1620/tjem.246.27 30210086

[17] Oyarzo C Bertoglia P Avendaño R Bacigalupo F Escudero A Acurio J . Adverse perinatal outcomes after the February 27th, 2010, Chilean earthquake. J Matern Fetal Neonatal Med. (2012) 25:1868–73. doi: 10.3109/14767058.2012.678437 22468878

[18] Pinkert M Dar S Goldberg D Abargel A Cohen-Marom O Kreiss Y . Lessons learned from an obstetrics and gynecology field hospital response to natural disasters. Obstet Gynecol. (2013) 122:532–6. doi: 10.1097/AOG.0b013e31829b5938 23921856

[19] Kyozuka H Ohhira T Murata T Yasuda S Fujimori K. Longitudinal effects of the Great East Japan Earthquake on obstetric outcomes. Life. (2023) 13:1–12. doi: 10.3390/life13081702 PMC10455406 37629559

[20] Sugawara J Iwama N Hoshiai T Tokunaga H Nishigori H Metoki H . Regional birth outcomes after the 2011 Great East Japan Earthquake and tsunami in Miyagi prefecture. Prehosp Disaster Med. (2018) 33:558–64. doi: 10.1017/S1049023X18000183 29560850

[21] Ulgu MM Birinci S Ensari TA Gözükara MG. Cesarean section rates in Turkey 2018–2023: overview of national data by using Robson ten group classification system. Turk J Obstet Gynecol. (2023) 20:191–8. doi: 10.4274/tjod.galenos.2023.68235 37667479 PMC10478731

[22] Eyi EGY Mollamahmutoglu L. An analysis of the high cesarean section rates in Turkey by Robson classification. J Matern Fetal Neonatal Med. (2021) 34:2682–92. doi: 10.1080/14767058.2019.1670806 31570019

[23] Dhital R Silwal RC Simkhada P van Teijlingen E Jimba M. Assessing knowledge and behavioural changes on maternal and newborn health among mothers following post-earthquake health promotion in Nepal. PLoS ONE. (2019) 14:e0220191. doi: 10.1371/journal.pone.0220191 31344147 PMC6657877

[24] Kedzierewicz R Couret A Cazes N Süsler A Arvieux C Arnaud I . Deployment of the French Civil Protection Field Hospital (ESCRIM) in Golbasi, Turkey, after the February 2023 earthquake: lessons learned. Prehosp Disaster Med. (2023) 38:522–8. doi: 10.1017/S1049023X23005873 37317865

[25] Menclova AK Stillman S. Maternal stress and birth outcomes: evidence from an unexpected earthquake swarm. Health Econ. (2020) 29:16–27. doi: 10.1002/hec.4162 32964531

[26] Tan CE Li HJ Zhang XG Zhang H Yu Han P An Q . The impact of the Wenchuan earthquake on birth outcomes. PLoS ONE. (2009) 4:e8200. doi: 10.1371/journal.pone.0008200 19997649 PMC2781160

[27] Tayfur I Bayramoglu B Simşek P Gunduz A. Medical response to the February 6, 2023, earthquakes in Hatay: challenges faced in the deadliest disaster in the history of Türkiye. Disaster Med Public Health Prep. (2024) 18:e45. doi: 10.1017/dmp.2024.21 38466322

[28] Yilmaz S Karakayali O Yilmaz S Çetin M Eroglu SE Dikme O . Emergency medicine association of Turkey disaster committee summary of field observations of the February 6th Kahramanmaraş earthquakes. Prehosp Disaster Med. (2023) 38:415–8. doi: 10.1017/S1049023X23000523 37198906

[29] Özşahin Z. Determinants of the desire to avoid pregnancy after the disaster of the century in Türkiye. BMC Womens Health. (2024) 24:496. doi: 10.1186/s12905-024-03330-6 39245745 PMC11382372

[30] Kurita N. Association of the Great East Japan earthquake and the Daiichi nuclear disaster in Fukushima City, Japan, with birth rates. JAMA Netw Open. (2019) 2:e187455. doi: 10.1001/jamanetworkopen.2018.7455 30681714 PMC6484541

[31] Lee DS Batyra E Castro A Wilde J. Human fertility after a disaster: a systematic literature review. Proc Biol Sci. (2023) 290:20230211. doi: 10.1098/rspb.2023.0211 37161332 PMC10170212

[32] Behrman JA Weitzman A. Effects of the 2010 Haiti earthquake on women's reproductive health. Stud Fam Plann. (2016) 47:3–17. doi: 10.1111/j.1728-4465.2016.00045.x 27027990

[33] Esfandyari M Vaghef-Mehrabany E Ebrahimi-Mameghani M. Varzaghan earthquake affected mothers' and their newborns' health more severely in socioeconomically vulnerable areas. Disaster Med Public Health Prep. (2018) 12:e96. doi: 10.1017/dmp.2018.96 30295234

[34] Suzuki K Yamagata Z Kawado M Hashimoto S. Effects of the Great East Japan earthquake on secondary sex ratio and perinatal outcomes. J Epidemiol. (2016) 26:76–83. doi: 10.2188/jea.JE20150055 26639751 PMC4728118

[35] Kyozuka H Murata T Yasuda S Ishii K Fujimori K Goto A . The effects of the Great East Japan earthquake on perinatal outcomes: results of the pregnancy and birth survey in the Fukushima health management survey. J Epidemiol. (2022) 32:S57–63. doi: 10.2188/jea.JE20210444 36464301 PMC9703925

[36] Kane JC Luitel NP Jordans MJD Kohrt BA Weissbecker I Tol WA. Mental health and psychosocial problems in the aftermath of the Nepal earthquakes: findings from a representative cluster sample survey. Epidemiol Psychiatr Sci. (2018) 27:301–10. doi: 10.1017/S2045796016001104 28065208 PMC5502203

[37] Cénat JM McIntee SE Blais-Rochette C. Symptoms of posttraumatic stress disorder, depression, anxiety and other mental health problems following the 2010 earthquake in Haiti: a systematic review and meta-analysis. J Affect Disord. (2020) 273:55–85. doi: 10.1016/j.jad.2020.04.046 32421623

[38] Bahmanjanbeh F Kohan S Yarmohammadian MH Haghshenas A. Evaluation of reproductive health indicators in women affected by the East Azarbaijan earthquake in August 2012. Iran J Nurs Midwifery Res. (2016) 21:504–9. doi: 10.4103/1735-9066.193414 27904635 PMC5114796

[39] Glynn LM Wadhwa PD Dunkel-Schetter C Chicz-DeMet A Sandman CA. When stress happens matters: effects of earthquake timing on stress responsivity in pregnancy. Am J Obstet Gynecol. (2001) 184:637–42. doi: 10.1067/mob.2001.111066 11262465

