# Supplementary material for: Disaster-related healthcare system disruption and maternal–neonatal outcomes
Source: Front Public Health. 2026 May 29;14:1784853. doi: 10.3389/fpubh.2026.1784853 (PMC13260484; doi:10.3389/fpubh.2026.1784853)
Supplement: Supplementary file 1 [file Table_1.docx]

| Supplementary Table 1. Clinical characteristics of the referred patients excluded from the analysis. | | | |
| --- | --- | --- | --- |
| Patient | **Reason for referral** | **Suspected condition** | **Outcome data** |
| 1 | Emergency transfer due to maternal instability | Suspected postpartum hemorrhage | Not available |
| 2 | Emergency transfer due to fetal indication | Suspected fetal distress | Not available |
| Outcome data were not available due to incomplete transfer records; therefore, the presence of severe outcomes such as complete uterine rupture or neonatal death cannot be definitively excluded. | | | |
